# Understanding and Targeting the Eukaryotic Translation Initiation Factor eIF4E in Head and Neck Cancer

**DOI:** 10.1155/2009/981679

**Published:** 2009-12-13

**Authors:** Biljana Culjkovic, Katherine L. Borden

**Affiliations:** Institute for Research in Immunology and Cancer and Department of Pathology and Cell Biology, Université de Montréal, Montreal, QC, Canada H4M 1J6

## Abstract

The eukaryotic translation initiation factor eIF4E is elevated in about 30% of human malignancies including HNSCC where its levels correlate with poor prognosis. Here, we discuss the biochemical and molecular underpinnings of the oncogenic potential of eIF4E. Studies in human leukemia specimens, and later in a mouse model of prostate cancer, strongly suggest that cells with elevated eIF4E develop an oncogene dependency to it, making them more sensitive to targeting eIF4E than normal cells. We describe several strategies that have been suggested for eIF4E targeting in the clinic: the use of a small molecule antagonist of eIF4E (ribavirin), siRNA or antisense oligonucleotide strategies, suicide gene therapy, and the use of a tissue-targeting 4EBP fusion peptide. The first clinical trial targeting eIF4E indicates that ribavirin effectively targets eIF4E in poor prognosis leukemia patients and more importantly leads to striking clinical responses including complete and partial remissions. Finally, we discuss the relevance of these findings to HNSCC.

## 1. Generalized Role for eIF4E in Cancer

The eukaryotic translation initiation factor 4E (eIF4E) is a protein that plays a central role in the regulation of gene expression at the posttranscriptional level. eIF4E binds the 7-methyl guanosine “m^7^G cap” structure found on the 5′ end of mRNAs. In the cytoplasm, eIF4E catalyses cap-dependent protein synthesis [1, 2]. Importantly, eIF4E effects the translation of some mRNAs, known as eIF4E sensitive, more than other transcripts. For instance, elevated eIF4E levels selectively increase translation of mRNAs coding for a variety of potent growth stimulatory proteins such as VEGF, Pim-1, and ornithine decarboxylase (ODC) [3–5]. In the nucleus, eIF4E mediates in the export of another subset of mRNAs (such as cyclin D1 and ODC mRNAs) to the cytoplasm [5–7]. Thus eIF4E can modulate gene expression at two levels: by exporting mRNAs to the cytoplasm increasing their concentration therein and by enhancing the translational efficiency of transcripts that are already in the cytoplasm. Not all transcripts are affected at both levels. Importantly, eIF4E requires its m^7^G cap binding function in order to act in either of these functions. Clearly, dysregulation of eIF4E will profoundly affect the cellular proteome.

The process of malignant transformation requires multiple molecular events involving activation of proto-oncogene products that stimulate growth and inactivation of suppressor genes that inhibit cellular proliferation. Together, these events result in selective dysregulation of cellular metabolism and growth. Critical control points in the cell cycle, DNA replication, and protein synthesis are just a few of many potential sites where alterations of normal functions may result in tumorigenesis. Because the overexpression of eIF4E results in the upregulation of multiple gene products that play critical roles in cycle progression and survival, it is not surprising that the elevation of eIF4E has been detected in various malignancies [3].

eIF4E is overexpressed in many epithelial cell cancers, including breast [8–12], colon [13, 14], bladder [15–19], cervix [20, 21], lung [22–24], and squamous cell carcinoma of the head and neck [25–32]. Some studies report that eIF4E is overexpressed in almost 100% of tumors of the breast, head and neck, and colon [8, 27, 32]. Several retrospective studies indicate that eIF4E elevation is correlated with poor prognosis. As discussed below, high eIF4E levels in the HNSCC correlated with higher incidence of relapse [26–29, 32]. eIF4E overexpression was detected at a range of 3–30 fold in breast carcinomas, compared to normal breast tissue [8, 10], and eIF4E levels were significantly increased in vascularized malignant ductules of invasive carcinomas [33]. Breast cancer patients with high eIF4E expression (>7-fold to normal) experienced a statistically significant poorer clinical outcome with a higher risk for recurrence and cancer related death [11]. Further, increased levels of eIF4E are observed in non-Hodgkin's lymphomas but not in benign lesions [34, 35]. Here, eIF4E levels correlated with the aggressiveness of these lesions [34, 35]. In prostate cancer, eIF4E levels were also correlated with worse prognosis [36]. In acute myeloid leukemia (AML), elevated eIF4E levels are characteristic of the poor prognosis M4 and M5 AML subtypes [37].

Given that both normal and cancer cells express eIF4E, it is important to develop therapeutic strategies that target cancer cells without harming normal cells. There is evidence that cancer cells have developed an oncogene addiction to, or dependency on, eIF4E. In studies in primary human leukemia specimens, subtypes of leukemias with elevated levels of eIF4E are sensitive to inhibition of eIF4E by antagonists at levels 100-fold less than those that effect normal bone marrow or other leukemic subtypes [38]. More recent studies suggest a similar case in a prostate cancer mouse model [39]. 

In animal models, eIF4E overexpression is correlated with not only increased numbers of tumors but also increased invasion, metastases, and angiogenesis [3, 15, 40]. Mice with transgene overexpression of eIF4E developed a variety of cancers of distinct histological origin [41]. These cancers develop despite the fact that the level of eIF4E overexpression in these mice is much less than the corresponding levels of eIF4E overexpression found in patients [32, 33, 37]. Further, a lymphoma mouse model showed that eIF4E overexpressing mice developed more lymphomas [42].

## 2. Dysregulation of eIF4E in HNSCC

eIF4E is found to be elevated in the vast majority (in some studies even 100% of cases) of HNSCC specimens, with levels being 3 to 24 fold elevated relative to normal controls [26–30, 32]. High eIF4E levels in surgical margins are predictive of increased risk of recurrence in HNSCC [26–29]. Overexpression of eIF4E in >5% of the basal layer of histologically tumor-free surgical margins of HNSCC patients predicted a significantly increased risk of recurrence [27]. This prediction is important for patient outcome as most HNSCC patients will succumb due to local recurrence [26, 28, 29]. It has been demonstrated that eIF4E overexpression is associated with eIF4E gene amplification in both HNSCC and in breast carcinomas [30, 43–45]. An increased level of eIF4E gene amplification was observed when benign tumors and invasive carcinomas of the head and neck were compared. Benign tumors only had moderate evidence for gene amplification, while malignant tumors had a 4–15 fold level of amplification [43]. eIF4E protein levels were elevated in premalignant lesions in the larynx, but to a lesser extent than observed in HNSCC [25]. These studies suggest that progression to the malignant phenotype paralleled eIF4E gene amplification and overexpression [43]. Also, there was a progressive increase in the degree of eIF4E gene amplification and protein expression when comparisons were made among samples from tumor free margins of resected carcinoma specimens, tumor free regions adjacent to tumor core and tumor core samples [44]. This suggests that molecular events such as eIF4E gene amplification may precede cellular morphological changes, and that surgical margins which appear tumor free microscopically, may have elevated eIF4E protein levels. Thus, eIF4E levels could be used as a marker for prediction of early recurrence. It has been postulated that somewhere in the multistep pathway of carcinogenesis, elevation of eIF4E is a necessary event in progression of most solid tumors, and that eIF4E does not only reflect the proliferative status of cells but also their malignant properties [28, 46]. 

**Figure 1:**
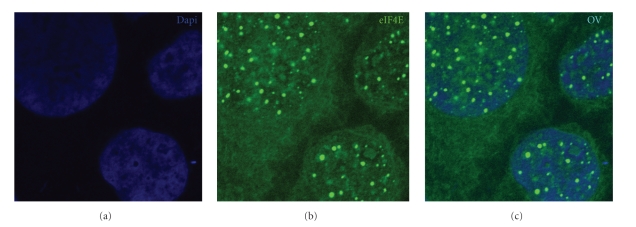
FaDu cells immunostained for eIF4E showing cytoplasmic and nuclear localization. Cells were stained using eIF4E mAb conjugated directly to FITC (green) and nuclear marker DAPI (blue) as described [37, 47]. Micrographs were collected on laser scanning confocal microscope using 100X objective and 2x digital zoom.

Consistent with their derivation from hypopharyngeal squamous carcinoma, FaDu cells [48] have elevated eIF4E [49], and as seen in many cell types, eIF4E is found in both the nucleus and cytoplasm (Figure 1). Further, eIF4E levels are elevated in FaDu cells due to both gene amplification, and increased mRNA stability [50]. Thus, there appears to be multiple ways to elevate eIF4E levels (see below).

## 3. Biochemical Underpinnings of eIF4E's Biological Effects

eIF4E overexpression profoundly alters the cellular proteome. However, experiments as early as 1980 [51] and more recent studies using knockdown strategies indicate that alterations in eIF4E expression do not uniformly alter the proteome [3, 5, 52–57]. In other words, the expression of some genes is more affected by modulation of eIF4E levels. These genes are referred to as eIF4E sensitive. In the cytoplasm, eIF4E recruits the transcript to the ribosome thereby increasing its translational efficiency. When eIF4E is overexpressed, sensitive transcripts have a higher ribosomes/mRNA ratio enabling more efficient translation without modulating mRNA levels in the cytoplasm. Notably, sensitive mRNAs have more highly structured 5′UTRs versus insensitive housekeeping mRNAs such as GAPDH or *β*-actin which contain short, unstructured 5′UTRs [3, 52, 58]. Transcripts controlled at this levels often code for proteins involved in proliferation such as c-Myc, Pim 1, VEGF, and ODC [4, 5, 55, 59]. 

Up to 68% of eIF4E is found in the nucleus of cells from a wide variety of species ranging from yeast to humans [7, 60–63]. These include FaDu cells, which have high eIF4E levels relative to normal cells and have eIF4E in both the nucleus and cytoplasm (Figure 1). As in the cytoplasm, only a subset of transcripts is sensitive to eIF4E dependant mRNA export [5]. These mRNAs contain a discrete 50 nucleotide element in their 3′UTR known as the eIF4E sensitivity element (4E-SE) [6, 64, 65]. Removal of the 4E-SE ablates eIF4E sensitivity [65]. Many mRNAs sensitive to eIF4E at the export level code for proteins that promote proliferation and survival. Increased export of the transcripts leads to increased levels of the mRNA available to the translation machinery, without altering translation efficiency [5]. In the nucleus, eIF4E is found in the nucleoplasm, in nuclear bodies co-localising with 4E-SE containing mRNAs or colocalising with promyelocytic leukaemia (PML) nuclear bodies (with no RNA). PML is a potent inhibitor of its mRNA export function and a potent inhibitor of eIF4E mediated transformation [47, 60, 66, 67]. 

**Figure 2:**
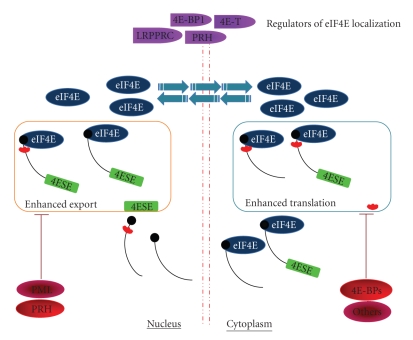
A diagram summarizing the nuclear and cytoplasmic functions of eIF4E. Some factors that directly regulate eIF4E functions and proteins involved in regulation of eIF4E subcellular distribution are shown. Not all regulators are shown for the sake of clarity. mRNAs are depicted as black lines with black balls denoting the 5′m^7^cap and with/without complex 5′UTRs shown in red and 4ESE element shown in green.

Regulation of transcripts by eIF4E can occur at the mRNA export level, the translation level, or both (Figure 2). For instance, cyclin D1 transcripts are only sensitive to eIF4E at the mRNA export level [5, 60, 61, 65]. VEGF transcripts are only sensitive to eIF4E at the level of translation [5, 58]. In contrast, ODC transcripts are sensitive to eIF4E at both the mRNA export and translation levels [5]. ODC is regulated at both levels because it contains both the complex 5′UTR sensitising it to translation and the 4E-SE in its 3′UTR sensitising it to eIF4E dependent mRNA export. Importantly, 4E-SE containing mRNAs is exported through a pathway that is distinct from bulk mRNA export [64]. Unlike bulk mRNA export which is TAP/NXF1 dependent, eIF4E dependent mRNA export is CRM1 dependent and requires the 4E-SE and the mRNA export factor LRPPRC [64, 68].

The combinatorial effects that eIF4E have on gene expression position it as a central node in an RNA regulon governing proliferation and cell survival [64, 65]. The RNA regulon is a theoretical construct that outlines a means by which posttranscriptional gene expression can be coordinated [69, 70]. In this model, elements in the UTRs of transcripts sensitise groups of transcripts to the same level of regulation. Transcripts with the same combination of elements, known as USER codes, will be coregulated. In this way, transcripts coding for proteins acting in the same biochemical pathway can have their production coordinated and thus the biochemical output of the pathway optimised. In the case of eIF4E, the complex 5′UTR and the 4E-SE in the 3′UTR can be considered to be USER codes for translation and export, respectively [6].

An example of the RNA regulon is the ability of eIF4E to modulate Akt signalling. eIF4E overexpression, via its mRNA export function, upregulates the expression of an activator of Akt, NBS1 [71, 72]. Furthermore, it enhances the expression of several downstream effectors of Akt including c-myc, cyclin D1, and cyclin E1 [5, 64]. eIF4E rescues serum-starved fibroblasts from serum-induced apoptosis. However, eIF4E loses this activity in Akt1^−/−^ cells whereas reintroduction of Akt1 enables eIF4E to rescue the cells again [71]. Thus, through the coordinated regulation of genes involved in the Akt pathway, eIF4E can promote cellular survival. These observations are particularly interesting in the context of HNSCC progression. In a study of HNSCC tumors and surgical margins, elevated levels of eIF4E correlated with elevated Akt activation [73].

In summary, eIF4E modulates gene expression at two levels: mRNA export and translation. These functions are coordinated through the RNA regulon. In many cases, eIF4E sensitive mRNAs act in the same biochemical pathways such as cell cycle progression or survival pathways. This coordination potently drives the oncogenic potential of eIF4E [6].

## 4. Molecular Basis for eIF4E Mediated Transformation

eIF4E overexpression leads to transformation in cell culture, as well as in animal models, as described above. Specifically, eIF4E overexpression leads to loss of contact inhibition of fibroblasts, growth in soft agar and increased proliferation [47, 58, 61, 74–76]. eIF4E overexpression rescues cells from certain types of apoptotic stimuli [77–81]. In fibroblasts, serum deprivation induced apoptotic rescue of eIF4E is Akt1 dependent [82]. Both the nuclear and cytoplasmic functions of eIF4E contribute to its oncogenic potential [37, 47, 58, 60, 61]. For instance, a mutant of eIF4E, W73A, which acts in mRNA export but is deficient in promotion of translation, acts in both transformation and survival to the same extent as wild-type eIF4E [47, 60, 76].

## 5. Redundant Regulation of eIF4E

Regulators of eIF4E functions are positioned to modulate the eIF4E regulon, co-ordinately modulating cell cycle progression, and cell survival (Figure 2). One of the best-characterized regulators of eIF4E is eIF4E binding protein 1 (BP1) [58, 83]. This protein uses a conserved eIF4E binding site to associate with eIF4E, and thereby precludes access of eIF4E to eIF4G and the rest of the translation machinery [58]. This binding site is defined as follows: YXXXXL*ϕ* (where X is any residue and *ϕ* is a hydrophobic residue). Studies suggest that BP1 increases cap affinity and thereby sequesters both eIF4E and the RNA in question from the translational machinery [1]. The association of BP1 with eIF4E is modulated by phosphorylation of BP1 [58, 83]. Phosphorylation of BP1 leads to a reduction in its interaction with eIF4E and thereby and results in increased translational activity of eIF4E. Phosphorylation is mTOR dependent and thus rapamycin treatment leads to reduced phosphorylation of BP1, increasing its association with eIF4E and thereby reducing translation of eIF4E sensitive mRNAs [84]. In contrast to eIF4E, BP1 overexpression sensitizes Ras transformed cells to apoptosis when treated with certain cytostatic drugs [85]. In addition, BP1 overexpression represses eIF4E mediated transformation of NIH 3T3 cells [58]. However, BP1^−/−^ and BP1^−/−^ BP2^−/−^ mice do not develop cancers more readily than controls [86–89], highlighting the importance of redundancy of regulators in the control of eIF4E. Studies on BP1 in the literature focus on BP1 as a regulator of the cytoplasmic functions of eIF4E [58, 84]. However, endogenous BP1 associates with eIF4E in both the nuclear and cytoplasmic compartments and thus likely modulates eIF4E activity at both the level of translation and mRNA export (see [90] and our unpublished observations). 

Counter-intuitively, BP1 levels are elevated in prostate and breast cancer and these levels correlate with a more advanced stage [91]. In esophageal cancers, there are more BP1-eIF4E complexes than in normal tissues, further complicating the accepted model of BP1 regulation of eIF4E [92]. Clearly, there is much more to be understood about BP1 and its implications for eIF4E activity.

There are many other regulators of eIF4E. The vast majority of these regulators contain the YXXXXL*ϕ* motif like eIF4G and the BPs. These regulators include a set of over 200 homeodomain proteins that contain this motif. Some of these members are negative regulators of eIF4E, such as PRH/Hex. PRH is a nuclear protein that impedes eIF4E's mRNA export function [76]. PRH overexpression leads to the cytoplasmic redistribution of eIF4E [37, 76]. Other members of this group of homeodomain containing regulators include Emx2, Otx, Engrailed 2, Hox11, Bicoid, and HoxA9 [93]. HoxA9 can stimulate both the nuclear and cytoplasmic functions of eIF4E [94]. Emx2 travels from one neuron to another through the synapse enabling localized translational control of eIF4E via signals to the adjacent neuron. In this way, Emx2 controls eIF4E activity remotely [93]**. **Thus, eIF4E function can be regulated in a tissue and context dependent manner.

There is also a discrete class of eIF4E regulators that utilize a RING domain to impede eIF4E function. These regulators include the promyelocytic leukemia protein PML, HHARI, and arenaviral Z proteins from LCMV and Lassa viruses [47, 60, 95]. PML and the Z proteins use their RING motifs to associate with eIF4E and inhibit eIF4E function by reducing the affinity of eIF4E for the m^7^G cap by up to 100-fold [28, 37, 96]. These were the first proteins reported to reduce the affinity of eIF4E for the m^7^G cap. PML is a key cellular inhibitor of the oncogenic activities of eIF4E. The ability to inhibit eIF4E function is closely tied with the ability of PML to impair cap binding, and thus the mRNA export activity of eIF4E [47, 64, 65, 71, 97]. Similarly, Z also impairs eIF4E cap binding and function [97]. Notably, PML and Z do not alter eIF4E levels, and therefore do not appear to act directly or indirectly in its protein stability, unlike other RINGs [97]. 

Clearly, the regulation of eIF4E activity is redundant and multifactorial. There are tissue specific regulators such as the homeodomain proteins and more ubiquitous regulators such as PML and BP1 (Figure 2). Redundancy of regulators is seen for both the nuclear and cytoplasmic arms of eIF4E activity.

## 6. Controlling eIF4E Localization—a Key Step in the Regulation of eIF4E

Clearly, modulating the subcellular distribution of eIF4E will have profound impacts on the sets of genes it regulates and thus on its biological effects. For instance, eIF4E localization is substantially altered during Xenopus gastrulation [7]. Furthermore, eIF4E nuclear-cytoplasmic localization changes dramatically during differentiation of mouse embryonic stem cells to macrophages (KLBB, unpublished observation). 

As discussed above, eIF4E is found in both the nuclear and cytoplasmic compartments. Recent studies indicate that within the cytoplasm, eIF4E is found not only associated with actively translating transcripts, but also with cytoplasmic structures known as processing bodies (P-bodies) [98, 99]. These structures contain a variety of factors including many associated with RNA degradation as well as eIF4E [100]. RNAs associated with these structures are sequestered from the translational machinery. It is thought that P-bodies are a temporary storage depot for these RNAs while their fate (in terms of degradation, sequestration, translation, etc.) is being decided [100]. Thus, in the cytoplasm, eIF4E is associated with both the translation machinery and in some cases with mRNAs that are being sequestered from this machinery (e.g., in P bodies), perhaps left there until the time is right for these mRNAs to be translated.

Ultimately, the biochemical pathways in which eIF4E functions (mRNA export, mRNA translation, or mRNA sequestration) depend on the subcellular distribution of eIF4E. Little is known about what regulates nuclear entry of eIF4E and what determines which cytoplasmic compartments in which eIF4E will be found. To date, the only factor known to directly modulate the subcellular distribution of eIF4E is the eIF4E transporter protein (4E-T) [98, 101]. 4E-T uses its conserved eIF4E binding site to interact directly with the dorsal surface of eIF4E. The original study suggested that 4E-T transported eIF4E protein into the nucleus [101]. However, several other studies, including subsequent studies by the Sonenberg group [98], indicate that overexpression of 4E-T leads to relocalization of the majority of nuclear eIF4E to the cytoplasm, where a subset is found in P-bodies. The molecular mechanism for this redistribution is not yet known. 

Other factors also modulate the subcellular distribution of eIF4E, including BP1 [90], the proline rich homeodomain protein PRH [37, 76], and the leucine rich protein LRPPRC [68] (Figure 2). PRH is a potent inhibitor of the mRNA export function of eIF4E [76]. PRH overexpression leads to redistribution of nuclear eIF4E to the cytoplasm [37, 76]. LRPPRC overexpression leads to re-distribution of eIF4E within the nucleus. Here, upon LRPPRC overexpression, LRPPRC competes for PML leading to reduced PML-eIF4E co-localization. This redistribution correlates with increased eIF4E dependent mRNA export [68]. In summary, these factors are positioned to impact the nuclear and cytoplasmic arms of eIF4E activity and thus alter the effects of eIF4E on the proteome.

There are other means to modulate the subcellular distribution of eIF4E. Interestingly, transduction of primary leukemia specimens (M4/M5 AML) with the inhibitor of NF*κ*B activity, I*κ*B-SR, leads to a substantial re-organization of eIF4E, reducing the amount of eIF4E found in the nuclear fraction and increasing the amount in the cytoplasm, and reorganization of the remaining eIF4E nuclear bodies into structures which are morphologically indistinguishable from normal cells [37, 93]. Thus, the subcellular distribution of eIF4E appears linked to NF*κ*B activity. As expected, transduction of I*κ*B-SR leads to reduced eIF4E dependent mRNA export in these specimens [37, 93]. In this way, eIF4E localization is linked to NFkB activity. 

In addition, the subcellular distribution of eIF4E can be modulated by small molecules [38, 47, 102]. Treatment of cells with the m^7^G cap analogue (m^7^GpppG) leads to disruption of eIF4E nuclear bodies and re-distribution of eIF4E to the cytoplasm [47, 102]. Treatment with a physical mimic of the m^7^G cap, ribavirin, has a similar effect where it leads to an increased fraction of eIF4E in the cytoplasm [38]. Consistently, ribavirin treatment leads to reduction in eIF4E dependent mRNA export. Note that ribavirin or m^7^GpppG, under these conditions, does not alter the levels of eIF4E [38, 47, 102]. 

In summary, factors such as 4E-T that so drastically affect the subcellular localization of eIF4E, are positioned to affect eIF4E's physiological activities in proliferation and oncogenic transformation (Figure 2). In addition, given that eIF4E modulates the expression of some transcripts only at one level (such as cyclin D1 at the export level or VEGF at the translation level), modulation of its subcellular distribution is likely to lead to differential effects on eIF4E sensitive transcripts. In this way, these eIF4E traffickers could modulate gene expression differentially favouring/disfavouring subsets of genes (e.g., export versus translation) and thereby modulate the biological effects of eIF4E. This level of modulation would allow a more tailored response to cellular stresses and stimuli.

## 7. How Does eIF4E Become Elevated in Cancer?

Given that elevated eIF4E levels are found in many human cancers and are associated with poor prognosis [28, 30, 43, 103], it is critical to understand how eIF4E levels become elevated. There are likely multiple mechanisms that could account for elevated eIF4E mRNA levels in these primary patient specimens, for example, gene amplification, transcriptional dysregulation, and alterations in mRNA stability. In fact, elevated eIF4E levels may result from any combination of these. For instance, eIF4E levels are elevated, at least in part, in breast cancer and head and neck squamous cell carcinomas due to amplification of the eIF4E gene [13, 104, 105]. Studies in cell culture indicate that the eIF4E promoter contains an E-box, and that its expression is regulated by c-myc [5, 6, 64]. Interestingly, c-myc is a downstream mRNA export and mRNA translation target of eIF4E which suggests existence of a potential feedback loop [106, 107]. As eIF4E is made in c-myc null mice, there must be other means by which it is induced [37]. Further, eIF4E mRNA levels are substantially reduced in primary leukemia specimens transduced with the I*κ*B-SR [3, 108]. In addition, some studies have found increased eIF4E expression during hypoxic conditions by IHC analysis of confined breast cancer biopsies [50]. In this way, the hypoxia that accompanies tumor growth may stimulate eIF4E expression.

Another mechanism that appears to be involved in the elevation of eIF4E in HNSCC is HuR dependent stabilization of eIF4E transcripts. Specifically, in FaDu cells, both HuR and eIF4E levels are elevated relative to control cells. Here, the mRNA stability factor, HuR, associates with eIF4E mRNA and enhances its stability [109]. HuR is a member of a family of proteins which modulate the stability of mRNAs by associating with U or AU rich elements (denoted AREs) typically in the 3′UTR of these messages [109]. Hu/ELAV family members are primarily neuronal with the exception of HuR, which is ubiquitously expressed. HuR modulates the expression of many proliferative mRNAs which contain AREs including (but not limited to): cyclin D1, cyclin B1, c-myc, VEGF, and so forth [109, 110]. Interestingly, many of these target mRNAs are also export and /or translational targets of eIF4E (e.g., all of the ones listed above). HuR has been implicated in oncogenesis. Its overexpression is correlated with the formation of tumors in mouse xenograft models [111, 112]. Microarray data of normal and cancer tissues indicated that HuR is elevated in human breast and lung cancer [96, 113, 114]. Further, HuR promotes angiogenesis, as does eIF4E [37]. The overlap in mRNA targets coupled to the fact that both eIF4E and HuR are involved in transformation and elevated in human cancers, suggests that eIF4E could be a downstream effector of HuR activity. Thus HuR is positioned to modulate the eIF4E regulon by both altering its expression and the expression of eIF4E's downstream effectors. Future studies that monitor HuR levels in HNSCC could be very interesting and may suggest HuR as another prognostic marker.

## 8. Targeting eIF4E in HNSCC- from Cells to Patients

To date, targeting of eIF4E in HNSCC remains in the preclinical stage. Three main pre-clinical strategies have been described: knockdown of eIF4E levels through the use of antisense oligonucleotides or RNA interference, suicide gene therapy, hormone analog—4EBP fusion peptide, and targeting eIF4E activity with ribavirin. 

Inhibition of eIF4E using antisense oligonucleotides to eIF4E was first performed in HeLa cells and Ras-transformed mouse fibroblasts, and resulted in the reversal of the malignant phenotype [115–117]. Decreasing levels of eIF4E in the human breast cancer cell line MDA-MB-435 and human prostate cancer cell line PC-3 diminished their angiogenic and tumorogenic properties [33, 39]. The first laboratory to find eIF4E levels elevated in both breast and HNSCC, the De Benedetti lab, was also the first to target eIF4E in HNSCC cells [33, 49]. Using antisense RNA to eIF4E, they demonstrated that they lowered both eIF4E levels and the levels of its downstream targets, VEGF and FGF-2. FaDu cells treated with antisense oligonucleotides also show reduced oncogenic properties of these cells including displaying increased contact inhibition, reduced growth in soft agar, and reduced tumorigenicity in xenograft mouse models [49]. A related strategy used small interfering RNAs targeting eIF4E either alone or in combination with cis-platin in the UMSCC22B HNSCC cell line [118]. As expected, siRNA to eIF4E lowered eIF4E levels and reduced the oncogenicity of this cell line. The addition of cis-platin increased the effects of the knockdown of eIF4E alone. This same strategy, combining siRNA to eIF4E with cis-platin, was also used in breast carcinoma cells with success [119]. 

Antisense oligonucleotides (ASOs) were also used by the Graff lab in a human prostate cancer xenograft mouse model [39]. Here, mice that intravenously received antisense oligonucleotides showed significant reduction of eIF4E expression and suppressed tumor growth. No toxicity was observed. The ASOs used also target murine eIF4E, leading to an 80% reduction of eIF4E in mouse liver; however there was no affect on body weight, organ weight, or liver transaminase levels. Eli Lilly is currently pursuing clinical trials using this strategy.

Suicide gene therapy is a method of introducing a gene, the expression of which will make a tumor cell uniquely susceptible to attack and destruction [120]. This strategy utilizes delivery of herpes simplex virus-thymidine kinase (HSV-Tk) by nonreplicative adenovirus vectors to the cells and subsequent ganciclovir (GCV) treatment [121, 122]. The HSV-Tk has the ability to phosphorylate and activate prodrug GCV to its cytotoxic triphosphate form with 1000-fold higher efficiency than its mammalian homologues. As a consequence, cells transfected with HSV-Tk can be targeted for death by treatment with ganciclovir, while normal cells would remain mainly unaffected [121, 122]. Although this strategy gained wide popularity as potential treatment for HNSCC, this strategy had two principal challenges: acceptable cytotoxic specificity to tumor cell targets and adequate delivery of the suicide gene. In order to specifically target eIF4E overexpressing cells, a long 5′UTR (from FGF-2) was fused to the thymidine kinase gene (5′UTR-Tk) to preferentially sensitize expression of this gene to eIF4E levels [123, 124]. This system was reported highly efficient in a broad spectrum of breast cancer cell lines [124]. Using a mouse minimal residual disease soft-tissue metastasis model for HNSCC, the Li group examined the efficacy of this strategy to target solid tumors cells that are overexpressing eIF4E [123]. In this study, mice that received Ad-HSV- 5′UTR-Tk fusion and GCV treatment showed longer disease free survival than the control group [123]. 

In order to inhibit eIF4E in ovarian cancers, Ko et al. [125] designed 4EBP-based peptide fused to an analog of gonadotropin-releasing hormone (GnRH) to specifically target ovarian and other endocrine cancer cells, that are widely overexpressing the GnRH receptor. This fusion peptide inhibited growth of the GnRH receptor expressing tumor cells and showed potent antitumor effect in a mouse xenograft model of epithelial ovarian cancer, without significant cytotoxic effects in other tissue.

## 9. Successful Targeting of eIF4E in the Clinic

To date, eIF4E has been successfully targeted only in a particularly aggressive form of acute myeloid leukemia French American British (FAB) subtype M4/M5 AML. These poor-prognosis leukemias are characterized by elevated eIF4E levels [37]. In these studies, ribavirin, a competitive inhibitor of the natural ligand of eIF4E the m^7^G cap, was used to target its biochemical and oncogenic activities [38]. In a phase II proof-of-principle clinical trial of refractory, relapsed or patients who cannot undergo induction chemotherapy were treated with ribavirin [126]. eIF4E inhibition led to striking clinical improvement including complete remission, partial remission, and blast response. Ribavirin was originally used as an antiviral drug and was well tolerated with no therapy related toxicities observed. Note that in these studies, ribavirin was the only cytotoxic chemotherapy permitted.

Clinical response correlated with inhibition of eIF4E activity and redistribution of the eIF4E protein from the nucleus to the cytoplasm [126]. In these AML patients, eIF4E was both highly upregulated and found mainly in the nucleus [38, 82]. The mRNA export activity of eIF4E is also upregulated in these specimens [37, 126]. After 28 days of treatment with ribavirin, eIF4E was markedly re-distributed from the nucleus to the cytoplasm [126]. Surprisingly, eIF4E protein levels were also downregulated, which is the first time this downregulation has been reported postribavirin treatment (note that in the previous experiments, ribavirin treatment was followed in cell culture for up to 48 hours, not 28 days as used for patients [127–130]). It is possible that this downregulation occurs via a negative feedback loop due to prolonged inhibition of eIF4E. Alternatively, decreased eIF4E levels could be a result of differential sensitivity within a heterogeneous cell population to ribavirin. At the same time, the production of eIF4E mRNA export targets such as cyclin D1 and NBS1 mRNA is repressed in patients. Further, eIF4E dependent Akt activation is reduced, which is consistent with its requirement for NBS1. 

Durability of clinical response is key to success. In the treatment of M4 and M5 AML, a regimen of chemotherapy typically combines Ara-C with danurubicin or idarubicin [127–130]. This regimen, named 7+3, induces remission in most patients. However, in the absence of consolidation therapy (typically with Ara-C), remissions only last 2–4 months [127–130]. Like many targeted monotherapies such as ATRA in APL or flt3 inhibitors in AML [131–134], after 2–4 months of ribavirin treatment, development of drug resistance was observed [126]. In these cases, although eIF4E levels remain low, eIF4E relocalizes to the nucleus, which generally correlated with relapse of the disease. The molecular events underpinning the return of eIF4E to the nucleus are not known, but clearly these events play a critical role in the response of these cells to ribavirin. To try to overcome resistance, ribavirin will be combined with other chemotherapy regimens. Although these studies are in AML patients, the ability to target eIF4E has clear implications for the development of treatments for HNSCC and other cancers with elevated eIF4E.

## 10. From Leukemia to HNSCC

Several previous studies indicate that ribavirin is an effective inhibitor of growth in FaDu cells [38, 82]. In xenograft mouse models, studies had shown that genetically reducing the levels of eIF4E protein by antisense RNA substantially impaired tumour growth [39, 119]. Similarly, addition of oral ribavirin to mice after FaDu xenograft led to significantly smaller tumours than for control animals [38]. Further, ribavirin inhibited anchorage dependent growth in FaDu cells in culture and significantly reduced levels of cyclin D1 and NBS1 proteins, and decreased Akt activation [82]. These findings in cell culture as well as results from treatment of AML patients suggest that targeting eIF4E with ribavirin in HNSCC may yield promising clinical results.

## 11. Conclusions

In this paper, we have described the biochemical function and biological effects of eIF4E. We summarize the roles and regulation of eIF4E in gene expression (Figure 2). We discussed the dysregulation of eIF4E in multiple cancers and the current strategies being considered to target its activity. eIF4E is an indicator of poor prognosis in HNSCC and hopefully, therapeutic approaches targeting eIF4E will benefit these patients.

## References

[1] von der Haar T, Gross JD, Wagner G, McCarthy JEG (2004). The mRNA cap-binding protein eIF4E in post-transcriptional gene expression. *Nature Structural and Molecular Biology*.

[2] Pestova TV, Hellen CUT (2000). The structure and function of initiation factors in eukaryotic protein synthesis. *Cellular and Molecular Life Sciences*.

[3] de Benedetti A, Harris AL (1999). eIF4E expression in tumors: its possible role in progression of malignancies. *International Journal of Biochemistry and Cell Biology*.

[4] Hoover DS, Wingett DG, Zhang J, Reeves R, Magnuson NS (1997). Pim-1 protein expression is regulated by its 5′-untranslated region and translation initiation factor elF-4E. *Cell Growth and Differentiation*.

[5] Rousseau D, Kaspar R, Rosenwald I, Gehrke L, Sonenberg N (1996). Translation initiation of ornithine decarboxylase and nucleocytoplasmic transport of cyclin D1 mRNA are increased in cells overexpressing eukaryotic initiation factor 4E. *Proceedings of the National Academy of Sciences of the United States of America*.

[6] Culjkovic B, Topisirovic I, Borden KLB (2007). Controlling gene expression through RNA regulons: the role of the eukaryotic translation initiation factor eIF4E. *Cell Cycle*.

[7] Strudwick S, Borden KLB (2002). The emerging roles of translation factor eIF4E in the nucleus. *Differentiation*.

[8] Kerekatte V, Smiley K, Hu B, Smith A, Gelder F, de Benedetti A (1995). The proto-oncogene/translation factor eIF4E: a survey of its expression in breast carcinomas. *International Journal of Cancer*.

[9] Li BDL, Gruner JS, Abreo F (2002). Prospective study of eukaryotic initiation factor 4E protein elevation and breast cancer outcome. *Annals of Surgery*.

[10] Li BDL, Liu L, Dawson M, de Benedetti A (1997). Overexpression of eukaryotic initiation factor 4E (eIF4E) in breast carcinoma. *Cancer*.

[11] Li BD, McDonald JC, Nassar R, de Benedetti A (1998). Clinical outcome in stage I to III breast carcinoma and eIF4E overexpression. *Annals of Surgery*.

[12] McClusky DR, Chu Q, Yu H (2005). A prospective trial on initiation factor 4E (elF4E) overexpression and cancer recurrence in node-positive breast cancer. *Annals of Surgery*.

[13] Rosenwald IB, Chen J-J, Wang S, Savas L, London IM, Pullman J (1999). Upregulation of protein synthesis initiation factor eIF-4E is an early event during colon carcinogenesis. *Oncogene*.

[14] Berkel HJ, Turbat-Herrera EA, Shi R, de Benedetti A (2001). Expression of the translation initiation factor eIF4E in the polyp-cancer sequence in the colon. *Cancer Epidemiology Biomarkers and Prevention*.

[15] Crew JP, Fuggle S, Bicknell R, Cranston DW, de Benedetti A, Harris AL (2000). Eukaryotic initiation factor-4E in superficial and muscle invasive bladder cancer and its correlation with vascular endothelial growth factor expression and tumour progression. *British Journal of Cancer*.

[16] Crew JP, O'Brien TS, Harris AL (1996). Bladder cancer angiogenesis, its role in recurrence, stage progression and as a therapeutic target. *Cancer and Metastasis Reviews*.

[17] Dickinson AJ, Fox SB, Persad RA, Hollyer J, Sibley GNA, Harris AL (1994). Quantification of angiogenesis as an independent predictor of prognosis in invasive bladder carcinomas. *British Journal of Urology*.

[18] Bochner BH, Cote RJ, Weidner N (1995). Angiogenesis in bladder cancer: relationship between microvessel density and tumor prognosis. *Journal of the National Cancer Institute*.

[19] Jaeger TM, Weidner N, Chew K (1995). Tumor angiogenesis correlates with lymph node metastases in invasive bladder cancer. *Journal of Urology*.

[20] Matthews-Greer J, Caldito G, de Benedetti A (2005). eIF4E as a marker for cervical neoplasia. *Applied Immunohistochemistry and Molecular Morphology*.

[21] Lee J-W, Choi J-J, Kyoung ML (2005). eIF-4E expression is associated with histopathologic grades in cervical neoplasia. *Human Pathology*.

[22] Rosenwald IB, Hutzler MJ, Wang S, Savas L, Fraire AE (2001). Expression of eukaryotic translation initiation factors 4E and 2*α* is increased frequently in bronchioloalveolar but not in squamous cell carcinomas of the lung. *Cancer*.

[23] Seki N, Takasu T, Mandai K (2002). Expression of eukaryotic initiation factor 4E in atypical adenomatous hyperplasia and adenocarcinoma of the human peripheral lung. *Clinical Cancer Research*.

[24] Jacobson BA, Alter MD, Kratzke MG (2006). Repression of cap-dependent translation attenuates the transformed phenotype in non-small cell lung cancer both in vitro and in vivo. *Cancer Research*.

[25] Chandy B, Abreo F, Nassar R, Stucker FJ, Nathan C-A (2002). Expression of the proto-oncogene eIF4E in inflammation of the oral cavity. *Otolaryngology: Head and Neck Surgery*.

[26] Nathan C-AO, Amirghahri N, Rice C, Abreo FW, Shi R, Stucker FJ (2002). Molecular analysis of surgical margins in head and neck squamous cell carcinoma patients. *Laryngoscope*.

[27] Nathan C-AO, Franklin S, Abreo FW, Nassar R, de Benedetti A, Glass J (1999). Analysis of surgical margins with the molecular marker eIF4E: a prognostic factor in patients with head and neck cancer. *Journal of Clinical Oncology*.

[28] Nathan C-AO, Liu L, Li BD, Abreo FW, Nandy I, de Benedetti A (1997). Detection of the proto-oncogene eIF4E in surgical margins may predict recurrence in head and neck cancer. *Oncogene*.

[29] Nathan C-AO, Sanders K, Abreo FW, Nassar R, Glass J (2000). Correlation of p53 and the proto-oncogene eIF4E in larynx cancers: prognostic implications. *Cancer Research*.

[30] Sorrells DL, Ghali GE, de Benedetti A, Nathan C-A, Li BDL (1999). Progressive amplification and overexpression of the eukaryotic initiation factor 4E gene in different zones of head and neck cancers. *Journal of Oral and Maxillofacial Surgery*.

[31] Franklin S, Pho T, Abreo FW (1999). Detection of the proto-oncogene eIF4E in larynx and hypopharynx cancers. *Archives of Otolaryngology: Head and Neck Surgery*.

[32] Nathan C-AO, Franklin S, Abreo FW (1999). Expression of eIF4E during head and neck tumorigenesis: possible role in angiogenesis. *Laryngoscope*.

[33] Nathan C-A, Carter P, Liu L (1997). Elevated expression of eIF4E and FGF-2 isoforms during vascularization of breast carcinomas. *Oncogene*.

[34] Wang S, Rosenwald IB, Hutzler MJ (1999). Expression of the eukaryotic translation initiation factors 4E and 2*α* in non-Hodgkin's lymphomas. *American Journal of Pathology*.

[35] Mossafa H, Damotte D, Jenabian A (2006). Non-Hodgkin's lymphomas with Burkitt-like cells are associated with c-Myc amplification and poor prognosis. *Leukemia and Lymphoma*.

[36] Graff JR, Konicek BW, Carter JH, Marcusson EG (2008). Targeting the eukaryotic translation initiation factor 4E for cancer therapy. *Cancer Research*.

[37] Topisirovic I, Guzman ML, McConnell MJ (2003). Aberrant eukaryotic translation initiation factor 4E-dependent mRNA transport impedes hematopoietic differentiation and contributes to leukemogenesis. *Molecular and Cellular Biology*.

[38] Kentsis A, Topisirovic I, Culjkovic B, Shao L, Borden KLB (2004). Ribavirin suppresses elF4E-mediated oncogenic transformation by physical mimicry of the 7-methyl guanosine mRNA cap. *Proceedings of the National Academy of Sciences of the United States of America*.

[39] Graff JR, Konicek BW, Vincent TM (2007). Therapeutic suppression of translation initiation factor eIF4E expression reduces tumor growth without toxicity. *Journal of Clinical Investigation*.

[40] Zimmer SG, de Benedetti A, Graff JR (2000). Translational control of malignancy: the mRNA cap-binding protein, eIF-4E, as a central regulator of tumor formation, growth, invasion and metastasis. *Anticancer Research*.

[41] Ruggero D, Montanaro L, Ma L (2004). The translation factor eIF-4E promotes tumor formation and cooperates with c-Myc in lymphomagenesis. *Nature Medicine*.

[42] Wendel H-G, de Stanchina E, Fridman JS (2004). Survival signalling by Akt and eIF4E in oncogenesis and cancer therapy. *Nature*.

[43] Haydon MS, Googe JD, Sorrells DS, Ghali GE, Li BDL (2000). Progression of eIF4E gene amplification and overexpression in benign and malignant tumors of the head and neck. *Cancer*.

[44] Sorrells DL, Black DR, Meschonat C (1998). Detection of eIF4E gene amplification in breast cancer by competitive PCR. *Annals of Surgical Oncology*.

[45] Sorrells DL, Meschonat C, Black D, Li BDL (1999). Pattern of amplification and overexpression of the eukaryotic initiation factor 4E gene in solid tumor. *Journal of Surgical Research*.

[46] Anthony B, Carter P, de Benedetti A (1996). Overexpression of the proto-oncogene/translation factor 4E in breast-carcinoma cell lines. *International Journal of Cancer*.

[47] Cohen N, Sharma M, Kentsis A, Perez JM, Strudwick S, Borden KLB (2001). PML RING suppresses oncogenic transformation by reducing the affinity of eIF4E for mRNA. *EMBO Journal*.

[48] Rangan SR (1972). A new human cell line (FaDu) from a hypopharyngeal carcinoma. *Cancer*.

[49] DeFatta RJ, Nathan C-AO, de Benedetti A (2000). Antisense RNA to eIF4E suppresses oncogenic properties of a head and neck squamous cell carcinoma cell line. *Laryngoscope*.

[50] Topisirovic I, Siddiqui N, Orolicki S (2009). Stability of eukaryotic translation initiation factor 4E mRNA is regulated by HuR, and this activity is dysregulated in cancer. *Molecular and Cellular Biology*.

[51] Sonenberg N, Trachsel H, Hecht S, Shatkin AJ (1980). Differential stimulation of capped mRNA translation in vitro by cap binding protein. *Nature*.

[52] Clemens MJ, Bommer U-A (1999). Translational control: the cancer connection. *International Journal of Biochemistry and Cell Biology*.

[53] Rosenwald IB, Kaspar R, Rousseau D (1995). Eukaryotic translation initiation factor 4E regulates expression of cyclin D1 at transcriptional and post-transcriptional levels. *Journal of Biological Chemistry*.

[54] Rosenwald IB, Lazaris-Karatzas A, Sonenberg N, Schmidt EV (1993). Elevated levels of cyclin D1 protein in response to increased expression of eukaryotic initiation factor 4E. *Molecular and Cellular Biology*.

[55] Kevil CG, de Benedetti A, Payne DK, Coe LL, Laroux FS, Alexander JS (1996). Translational regulation of vascular permeability factor by eukaryotic initiation factor 4E: implications for tumor angiogenesis. *International Journal of Cancer*.

[56] Shantz LM, Hu R-H, Pegg AE (1996). Regulation of ornithine decarboxylase in a transformed cell line that overexpresses translation initiation factor eIF-4E. *Cancer Research*.

[57] Shantz LM, Pegg AE (1994). Overproduction of ornithine decarboxylase caused by relief of translational repression is associated with neoplastic transformation. *Cancer Research*.

[58] Sonenberg N, Gingras A-C (1998). The mRNA 5′ cap-binding protein elF4E and control of cell growth. *Current Opinion in Cell Biology*.

[59] Rhoads RE, Joshi-Barve S, Rinker-Schaeffer C (1993). Mechanism of action and regulation of protein synthesis initiation factor 4E: effects on mRNA discrimination, cellular growth rate, and oncogenesis. *Progress in Nucleic Acid Research and Molecular Biology*.

[60] Lai H-K, Borden KLB (2000). The promyelocytic leukemia (PML) protein suppresses cyclin D1 protein production by altering the nuclear cytoplasmic distribution of cyclin D1 mRNA. *Oncogene*.

[61] Topisirovic I, Capili AD, Borden KLB (2002). Gamma interferon and cadmium treatments modulate eukaryotic initiation factor 4E-dependent mRNA transport of cyclin D1 in a PML-dependent manner. *Molecular and Cellular Biology*.

[62] Iborra FJ, Jackson DA, Cook PR (2001). Coupled transcription and translation within nuclei of mammalian cells. *Science*.

[63] Lejbkowicz F, Goyer C, Darveau A, Neron S, Lemieux R, Sonenberg N (1992). A fraction of the mRNA 5′ cap-binding protein, eukaryotic initiation factor 4E, localizes to the nucleus. *Proceedings of the National Academy of Sciences of the United States of America*.

[64] Culjkovic B, Topisirovic I, Skrabanek L, Ruiz-Gutierrez M, Borden KLB (2006). eIF4E is a central node of an RNA regulon that governs cellular proliferation. *Journal of Cell Biology*.

[65] Culjkovic B, Topisirovic I, Skrabanek L, Ruiz-Gutierrez M, Borden KLB (2005). eIF4E promotes nuclear export of cyclin D1 mRNAs via an element in the 3′UTR. *Journal of Cell Biology*.

[66] Borden KLB (2008). Pondering the puzzle of PML (promyelocytic leukemia) nuclear bodies: can we fit the pieces together using an RNA regulon?. *Biochimica et Biophysica Acta*.

[67] Scheper GC, Parra JL, Wilson M (2003). The N and C termini of the splice variants of the human mitogen-activated protein kinase-interacting kinase Mnk2 determine activity and localization. *Molecular and Cellular Biology*.

[68] Topisirovic I, Siddiqui N, Lapointe VL (2009). Molecular dissection of the eukaryotic initiation factor 4E (eIF4E) export-competent RNP. *EMBO Journal*.

[69] Keene JD (2007). RNA regulons: coordination of post-transcriptional events. *Nature Reviews Genetics*.

[70] Keene JD, Lager PJ (2005). Post-transcriptional operons and regulons co-ordinating gene expression. *Chromosome Research*.

[71] Culjkovic B, Tan K, Orolicki S, Amri A, Meloche S, Borden KLB (2008). The eIF4E RNA regulon promotes the Akt signaling pathway. *Journal of Cell Biology*.

[72] Chen Y-C, Su Y-N, Chou P-C (2005). Overexpression of NBS1 contributes to transformation through the activation of phosphatidylinositol 3-kinase/Akt. *Journal of Biological Chemistry*.

[73] Nathan C-AO, Amirghahari N, Abreo F (2004). Overexpressed eIF4E is functionally active in surgical margins of head and neck cancer patients via activation of the Akt/mammalian target of rapamycin pathway. *Clinical Cancer Research*.

[74] Lazaris-Karatzas A, Montine KS, Sonenberg N (1990). Malignant transformation by a eukaryotic initiation factor subunit that binds to mRNA 5′ cap. *Nature*.

[75] de Benedetti A, Graff JR (2004). eIF-4E expression and its role in malignancies and metastases. *Oncogene*.

[76] Topisirovic I, Culjkovic B, Cohen N, Perez JM, Skrabanek L, Borden KLB (2003). The proline-rich homeodomain protein, PRH, is a tissue-specific inhibitor of eIF4E-dependent cyclin D1 mRNA transport and growth. *EMBO Journal*.

[77] Li S, Perlman DM, Peterson MS (2004). Translation initiation factor 4E blocks endoplasmic reticulum-mediated apoptosis. *Journal of Biological Chemistry*.

[78] Li S, Takasu T, Perlman DM (2003). Translation factor eIF4E rescues cells from Myc-dependent apoptosis by inhibiting cytochrome c release. *Journal of Biological Chemistry*.

[79] Polunovsky VA, Gingras A-C, Sonenberg N (2000). Translational control of the antiapoptotic function of Ras. *Journal of Biological Chemistry*.

[80] Polunovsky VA, Rosenwald IB, Tan AT (1996). Translational control of programmed cell death: eukaryotic translation initiation factor 4e blocks apoptosis in growth-factor-restricted fibroblasts with physiologically expressed or deregulated Myc. *Molecular and Cellular Biology*.

[81] Tan A, Bitterman P, Sonenberg N, Peterson M, Polunovsky V (2000). Inhibition of Myc-dependent apoptosis by eukaryotic translation initiation factor 4E requires cyclin D1. *Oncogene*.

[82] Tan K, Culjkovic B, Amri A, Borden KLB (2008). Ribavirin targets eIF4E dependent Akt survival signaling. *Biochemical and Biophysical Research Communications*.

[83] Gingras A-C, Gygi SP, Raught B (1999). Regulation of 4E-BP1 phosphorylation: a novel two step mechanism. *Genes and Development*.

[84] Proud CG (2007). Signalling to translation: how signal transduction pathways control the protein synthetic machinery. *Biochemical Journal*.

[85] Li S, Sonenberg N, Gingras A-C (2002). Translational control of cell fate: availability of phosphorylation sites on translational repressor 4E-BP1 governs its proapoptotic potency. *Molecular and Cellular Biology*.

[86] Blackshear PJ, Stumpo DJ, Carballo E, Lawrence JC (1997). Disruption of the gene encoding the mitogen-regulated translational modulator PHAS-I in mice. *Journal of Biological Chemistry*.

[87] Banko JL, Hou L, Poulin F, Sonenberg N, Klann E (2006). Regulation of eukaryotic initiation factor 4E by converging signaling pathways during metabotropic glutamate receptor-dependent long-term depression. *Journal of Neuroscience*.

[88] Le Bacquer O, Petroulakis E, Paglialunga S (2007). Elevated sensitivity to diet-induced obesity and insulin resistance in mice lacking 4E-BP1 and 4E-BP2. *Journal of Clinical Investigation*.

[89] Tsukiyama-Kohara K, Poulin F, Kohara M (2001). Adipose tissue reduction in mice lacking the translational inhibitor 4E-BP1. *Nature Medicine*.

[90] Rong L, Livingstone M, Sukarieh R (2008). Control of eIF4E cellular localization by eIF4E-binding proteins, 4E-BPs. *RNA*.

[91] Armengol G, Rojo F, Castellvi J (2007). 4E-binding protein 1: a key molecular “funnel factor” in human cancer with clinical implications. *Cancer Research*.

[92] Salehi Z, Mashayekhi F (2006). Expression of the eukaryotic translation initiation factor 4E (eIF4E) and 4E-BP1 in esophageal cancer. *Clinical Biochemistry*.

[93] Topisirovic I, Borden KLB (2005). Homeodomain proteins and eukaryotic translation initiation factor 4E (elF4E): an unexpected relationship. *Histology and Histopathology*.

[94] Topisirovic I, Kentsis A, Perez JM, Guzman ML, Jordan CT, Borden KLB (2005). Eukaryotic translation initiation factor 4E activity is modulated by HOXA9 at multiple levels. *Molecular and Cellular Biology*.

[95] Ardley HC, Tan NGS, Rose SA, Markham AF, Robinson PA (2001). Features of the parkin/ariadne-like ubiquitin ligase, HHARI, that regulate its interaction with the ubiquitin-conjugating enzyme, Ubch7. *Journal of Biological Chemistry*.

[96] Graff JR, Zimmer SG (2003). Translational control and metastatic progression: enhanced activity of the mRNA cap-binding protein eIF-4E selectively enhances translation of metastasis-related mRNAs. *Clinical and Experimental Metastasis*.

[97] Kentsis A, Dwyer EC, Perez JM (2001). The RING domains of the promyelocytic leukemia protein PML and the arenaviral protein Z repress translation by directly inhibiting translation initiation factor eIF4E. *Journal of Molecular Biology*.

[98] Ferraiuolo MA, Basak S, Dostie J, Murray EL, Schoenberg DR, Sonenberg N (2005). A role for the eIF4E-binding protein 4E-T in P-body formation and mRNA decay. *Journal of Cell Biology*.

[99] Parker R, Sheth U (2007). P bodies and the control of mRNA translation and degradation. *Molecular Cell*.

[100] Anderson P, Kedersha N (2006). RNA granules. *Journal of Cell Biology*.

[101] Dostie J, Ferraiuolo M, Pause A, Adam SA, Sonenberg N (2000). A novel shuttling protein, 4E-T, mediates the nuclear import of the mRNA 5′ cap-binding protein, eIF4E. *EMBO Journal*.

[102] Dostie J, Lejbkowicz F, Sonenberg N (2000). Nuclear eukaryotic initiation factor 4E (eIF4E) colocalizes with splicing factors in speckles. *Journal of Cell Biology*.

[103] Sorrells DL, Ghali GE, Meschonat C (1999). Competitive PCR to detect elF4E gene amplification in head and neck cancer. *Head and Neck*.

[104] Schmidt EV (2004). The role of c-Myc in regulation of translation initiation. *Oncogene*.

[105] Lin C-J, Cencic R, Mills JR, Robert F, Pelletier J (2008). c-Myc and eIF4F are components of a feedforward loop that links transcription and translation. *Cancer Research*.

[106] Bush A, Mateyak M, Dugan K (1998). c-Myc null cells misregulate cad and gadd45 but not other proposed c-Myc targets. *Genes and Development*.

[107] Ravitz MJ, Chen L, Lynch M, Schmidt EV (2007). C-Myc repression of TSC2 contributes to control of translation initiation and Myc-induced transformation. *Cancer Research*.

[108] DeFatta RJ, Turbat-Herrera EA, Li BDL, Anderson W, de Benedetti A (1999). Elevated expression of eIF4E in confined early breast cancer lesions: possible role of hypoxia. *International Journal of Cancer*.

[109] Lopez de Silanes I, Lal A, Gorospe M (2005). HuR: post-transcriptional paths to malignancy. *RNA Biology*.

[110] Lopez de Silanes I, Fan J, Yang X (2003). Role of the RNA-binding protein HuR in colon carcinogenesis. *Oncogene*.

[111] Denkert C, Weichert W, Pest S (2004). Overexpression of the embryonic-lethal abnormal vision-like protein HuR in ovarian carcinoma is a prognostic factor and is associated with increased cyclooxygenase 2 expression. *Cancer Research*.

[112] Denkert C, Winzer K-J, Hauptmann S (2004). Prognostic impact of cyclooxygenase-2 in breast cancer. *Clinical Breast Cancer*.

[113] Houseley J, LaCava J, Tollervey D (2006). RNA-quality control by the exosome. *Nature Reviews Molecular Cell Biology*.

[114] Yoo PS, Mulkeen AL, Cha CH (2006). Post-transcriptional regulation of vascular endothelial growth factor: implications for tumor angiogenesis. *World Journal of Gastroenterology*.

[115] Rinker-Schaeffer CW, Graff JR, de Benedetti A, Zimmer SG, Rhoads RE (1993). Decreasing the level of translation initiation factor 4E with antisense RNA causes reversal of ras-mediated transformation and tumorigenesis of cloned rat embryo fibroblasts. *International Journal of Cancer*.

[116] de Benedetti A, Joshi-Barve S, Rinker-Schaeffer C, Rhoads RE (1991). Expression of antisense RNA against initiation factor eIF-4E mRNA in HeLa cells results in lengthened cell division times, diminished translation rates, and reduced levels of both eIF-4E and the p220 component of eIF-4F. *Molecular and Cellular Biology*.

[117] Graff JR, Boghaert ER, de Benedetti A (1995). Reduction of translation initiation factor 4E decreases the malignancy of ras-transformed cloned rat embryo fibroblasts. *International Journal of Cancer*.

[118] Oridate N, Kim H-J, Xu X, Lotan R (2005). Growth inhibition of head and neck squamous carcinoma cells by small interfering RNAs targeting eIF4E or cyclin D1 alone or combined with cisplatin. *Cancer Biology and Therapy*.

[119] Dong K, Wang R, Wang X (2009). Tumor-specific RNAi targeting eIF4E suppresses tumor growth, induces apoptosis and enhances cisplatin cytotoxicity in human breast carcinoma cells. *Breast Cancer Research and Treatment*.

[120] Xi S, Grandis JR (2003). Gene therapy for the treatment of oral squamous cell carcinoma. *Journal of Dental Research*.

[121] O'Malley BW, Chen S-H, Schwartz MR, Woo SLC (1995). Adenovirus-mediated gene therapy for human head and neck squamous cell cancer in a nude mouse model. *Cancer Research*.

[122] O'Malley BW, Cope KA, Chen S-H, Li D, Schwartz MR, Woo SLC (1996). Combination gene therapy for oral cancer in a murine model. *Cancer Research*.

[123] Siegele B, Cefalu C, Holm N (2008). eIF4E-targeted suicide gene therapy in a minimal residual mouse model for metastatic soft-tissue head and neck squamous cell carcinoma improves disease-free survival. *Journal of Surgical Research*.

[124] Mathis JM, Williams BJ, Sibley DA (2006). Cancer-specific targeting of an adenovirus-delivered herpes simplex virus thymidine kinase suicide gene using translational control. *Journal of Gene Medicine*.

[125] Song YK, Guo H, Barengo N, Naora H (2009). Inhibition of ovarian cancer growth by a tumor-targeting peptide that binds eukaryotic translation initiation factor 4E. *Clinical Cancer Research*.

[126] Assouline S, Culjkovic B, Cocolakis E (2009). Molecular targeting of the oncogene eIF4E in acute myeloid leukemia (AML): a proof-of-principle clinical trial with ribavirin. *Blood*.

[127] Brandwein JM, Gupta V, Schuh AC (2008). Predictors of response to reinduction chemotherapy for patients with acute myeloid leukemia who do not achieve complete remission with frontline induction chemotherapy. *American Journal of Hematology*.

[128] Camera A, Rinaldi CR, Palmieri S (2009). Sequential continuous infusion of fludarabine and cytarabine associated with liposomal daunorubicin (DaunoXome) (FLAD) in primary refractory or relapsed adult acute myeloid leukemia patients. *Annals of Hematology*.

[129] Martin MG, Abboud CN (2008). Induction therapy for elderly patients with acute myeloid leukemia. *Blood Reviews*.

[130] Ruiz-Arguelles GJ, Apreza-Molina MaG (1997). Results of the treatment of acute leukemia in adolescents. *Revista de Investigacion Clinica*.

[131] Castaigne S, Degos L (1995). Treatment of acute promyelocytic leukemia by all trans retinoic acid. *Comptes Rendus des Seances de la Societe de Biologie et de ses Filiales*.

[132] Avvisati G, Tallman MS (2003). All-trans retinoic acid in acute promyelocytic leukaemia. *Best Practice and Research: Clinical Haematology*.

[133] Sawyers CL (2002). Finding the next Gleevec: FLT3 targeted kinase inhibitor therapy for acute myeloid leukemia. *Cancer Cell*.

[134] Knapper S, Burnett AK, Littlewood T (2006). A phase 2 trial of the FLT3 inhibitor lestaurtinib (CEP701) as first-line treatment for older patients with acute myeloid leukemia not considered fit for intensive chemotherapy. *Blood*.

